# Bioprospecting of Natural Products from Medicinal Plants

**DOI:** 10.3390/plants13243556

**Published:** 2024-12-20

**Authors:** Maria João Rodrigues

**Affiliations:** Centre of Marine Sciences (CCMAR/CIMAR LA), Faculty of Sciences and Technology, University of Algarve, Ed. 7, Campus of Gambelas, 8005-139 Faro, Portugal; mjrodrigues@ualg.pt

The exploration of natural products derived from medicinal plants that provide an abundance of bioactive compounds has long been a cornerstone of scientific discovery, revolutionizing fields such as medicine, agriculture, and biotechnology [1]. Notable examples include artemisinin, a life-saving antimalarial derived from *Artemisia annua* [2], and taxol, a chemotherapeutic agent sourced from the Pacific yew tree [3], both of which highlight the immense potential of medicinal plants to address global health challenges. Over the past decade, rapid advancements in analytical techniques, omics technologies, and bioinformatics have significantly improved our ability to uncover and characterize these natural products. Despite these strides, critical challenges persist in the field, including the sustainable utilization of plant resources, the discovery of novel compounds with unique modes of action, and the translation of these findings into practical, real-world applications.

This Special Issue, Bioprospecting of Natural Products from Medicinal Plants, highlights the vast potential of medicinal plants as reservoirs of bioactive compounds. Comprising twelve articles—an inspiring blend of original research and review papers—this collection exemplifies the collaborative spirit of researchers across Europe, Asia, Africa, and the Americas. The global significance of medicinal plant research is further emphasized by the diverse contributions, which reflect the unique biodiversity and ecological challenges faced by different regions. Such international collaboration is vital, as each country brings its distinctive resources and expertise to uncovering and harnessing new bioactive compounds.

Several studies emphasize the phytochemical and biological characterization of medicinal plants. Bendjedou et al. investigated *Lycium intricatum*, revealing its antioxidant and enzyme inhibitory properties related to neurological diseases, type-2 diabetes mellitus, obesity, and skin hyperpigmentation [4]. Similarly, Rodrigues et al. explored the nutritional and therapeutic potential of *Cladium mariscus* seeds, identifying their value as sources of bioactive ingredients to treat neurological diseases, type-2 diabetes mellitus, obesity, and skin hyperpigmentation [5]. Youssef et al. examined *Limonium spathulatum*, identifying enzyme inhibitors and phenolic compounds with potential applications in the health and food industries, such as treating neurological diseases, type-2 diabetes mellitus, obesity, and skin hyperpigmentation, as well as preventing food browning [6]. Mao et al. demonstrated the antiviral efficacy of extracts from *Hedyotis diffusa* and *Artemisia capillaris* against emerging viral pathogens, namely dengue and Zika viruses, underlining their potential in public health applications [7]. Baz et al. tested the efficacy of *Araucaria heterophylla* and *Commiphora molmol* extracts against camel and cattle blood-sucking ectoparasites, presenting promising results for veterinary use [8]. Song et al. studied the anti-inflammatory functions of methanol extracts from *Malus baccata* leaves and shoots, identifying potential uses in managing inflammatory diseases [9]. Innovative cultivation and extraction methodologies also featured prominently in the research. Iqbal et al. presented a nano-integrated plant tissue culture approach to enhance curcuminoid production in *Curcuma longa* callus cultures, offering sustainable production strategies [10].

In addition to these studies, two review papers provided comprehensive insights into medicinal plants. Kamal et al. conducted an extensive review on the medicinal properties, phytochemistry, and pharmacological applications of *Vitex* species, highlighting their therapeutic potential across various health conditions [11]. While Indradi et al. explored traditional medicinal plants from Papua Island (Indonesia), uncovering anti-plasmodial agents and emphasizing the pharmacological potential of the region’s unique flora [12].

Among these contributions, three papers stood out as the Editor’s Choice for their exceptional innovation and impact. Park et al. evaluated the effects of *Allium hookeri* extracts on hair-inductive properties in human dermal papilla cells, suggesting applications for hair regeneration therapies [13]. Rodrigues et al. emphasized the bioactivity of *Frankenia laevis*, focusing on its ability to address type-2 diabetes and its cytotoxic properties against hepatocarcinoma cells [14]. Lee et al. determined the anti-aging properties of *Potentilla paradoxa*, highlighting its potential in skincare formulations [15].

Moreover, this Special Issue also highlights several future directions for the field. Interdisciplinary approaches integrating omics technologies, machine learning, and biotechnological tools will be essential for unlocking the full potential of medicinal plants. Furthermore, addressing sustainability challenges and ensuring biodiversity conservation remain pivotal for ethical and effective bioprospecting.

Despite these advancements, the field of natural product research from medicinal plants is at a turning point. Key challenges remain, particularly in understanding the ecological roles of bioactive compounds and their biosynthesis under varied environmental conditions. Translating laboratory findings into commercially viable products also demands robust interdisciplinary collaboration and substantial investment. Future research should prioritize exploring underutilized and extreme environments, such as saline or arid regions, to uncover unique bioactive compounds. Integrating omics technologies—genomics, transcriptomics, metabolomics, and proteomics—is vital for decoding biosynthetic pathways and enhancing compound production. Equally important is the conservation of biodiversity through collaborative initiatives that engage researchers, policymakers, and local communities to ensure sustainable and equitable bioprospecting. Strengthening preclinical and clinical pipelines will facilitate the development of plant-derived therapeutics, while leveraging AI and big data can accelerate candidate identification and uncover complex metabolomic interactions.

As we conclude this Special Issue, it is evident that the field of bioprospecting from medicinal plants is entering a transformative phase. The contributions presented herein provide a foundation for tackling some of the most pressing challenges and seizing emerging opportunities in this field. By fostering interdisciplinary collaborations and embracing technological advancements, the scientific community is well-positioned to unlock the full potential of natural products for the benefit of society and the planet.

We extend our gratitude to all the authors, reviewers, and editorial staff who have contributed to the success of this Special Issue. We look forward to witnessing how the insights and innovations presented here will shape the future of natural product research and inspire the next generation of scientific breakthroughs.
